# Neoadjuvant chemoradiotherapy followed by esophagectomy vs. surgery alone in the treatment of resectable esophageal squamous cell carcinoma

**DOI:** 10.3892/mco.2013.128

**Published:** 2013-05-21

**Authors:** YOSHINORI FUJIWARA, REIGETSU YOSHIKAWA, NORIHIKO KAMIKONYA, TSUYOSHI NAKAYAMA, KOTARO KITANI, MASANORI TSUJIE, MASAO YUKAWA, JOHJI HARA, TAKEHIRA YAMAMURA, MASATOSHI INOUE

**Affiliations:** 1 Department of Digestive Surgery, Nara Hospital, Kinki University School of Medicine, Ikoma, Nara 630-0293;; 2 Department of Surgery, Kanzaki Hospital, Amagasaki, Hyogo 661-0967;; 3 Departments of Radiology, Nishinomiya, Hyogo 663-8501, Japan; 4 Surgery, Hyogo College of Medicine, Nishinomiya, Hyogo 663-8501, Japan

**Keywords:** esophageal cancer, trimodality therapy, survival

## Abstract

In order to improve the survival of esophageal cancer patients, a trimodality therapy consisting of esophagectomy in combination with neoadjuvant chemoradiotherapy (CRT) has been developed. In this study, we evaluated whether neoadjuvant CRT improved the outcomes of patients with resectable esophageal squamous cell carcinoma (ESCC) compared to surgery alone. Eighty-eight patients with resectable ESCC were treated with either neoadjuvant CRT followed by surgical resection (Group A, n=52), or surgery alone (Group B, n=36). CRT consisted of 5-fluorouracil (5-FU, 500 mg/m^2^ on days 1–5) and cisplatin (CDDP, 10–20 mg/kg body weight on days 1–5), repeated after 3 weeks. Survival analysis was performed using the log-rank test with the Kaplan-Meier method. The clinical response of the primary tumor and metastatic nodes was 80.8%. The postoperative complications profile was similar between the two groups, except for anastomotic leakage. The median survival time (MST) was not reached in Group A and was 27.4 months in Group B. The estimated 5-year overall survival (OS) rate was 50.3% in Group A and 39.9% in Group B (P=0.134). As regards stage II/III disease, Group A exhibited a better disease-free survival (DFS) compared to Group B (5-year DFS: 57.2% in Group A vs. 31.4% in Group B; P=0.025). Simultaneous locoregional and distant recurrences were more common in the surgery alone group (Group B, P=0.047). Neoadjuvant CRT with 5-FU and CDDP did not contribute to a better prognosis in patients with resectable ESCC. However, it may be beneficial for patients with stage II/III disease.

## Introduction

Esophageal cancer is the eighth most common type of cancer worldwide, with an estimated 481,645 new cases in 2008, and the sixth most common cause of cancer-related mortality, with 406,533 deaths in 2008 (1). Esophageal squamous cell carcinomas (ESCCs) are more common in Asian countries, including Japan, compared to Western countries, where adenocarcinomas in the lower third of the esophagus are commonly encountered. According to the National Cancer Center data base (2), 11,746 Japanese patients succumbed to esophageal cancer in 2008, which was equivalent to 3.26% of the total deaths from malignant neoplasms in Japan.

Esophageal cancer is highly aggressive and has a poor prognosis due to early metastasis to the lymph nodes, as well as metastasis to distant organs (3–5). Surgery has been considered as the mainstay of treatment for patients with confirmed, locoregionally confined esophageal carcinoma. However, until a decade ago the 3- or 5-year survival rate was <30% worldwide, according to previous studies using several different approaches (6,7–10). In Japan, the survival rate has shown improvement over the last two decades since three-field lymphadenectomy was introduced in the early 1980s by Isono *et al* (11) and Ando *et al* (12), a modality that is now widely used. In comparison, en bloc esophagectomy, also known as extensive esophagectomy, is performed in Western countries (13–15). However, even with extensive radical surgery involving the esophagus, locoregional or distant recurrences have been observed in 8–32% of patients (16–25). The estimated 5-year survival rates for esophageal cancer treated with curative intent were 34–53% (11,26–28). To improve the survival of locoregional esophageal carcinoma, multimodal therapy comprising chemotherapy and/or radiotherapy in combination with radical surgery has been developed. An approach involving neoadjuvant chemoradiotherapy (CRT) followed by esophagectomy, known as trimodality therapy (29,30), is mainly used. It offers the potential advantages of tumor downstaging, reduced dissemination of malignant cells during surgery and prevention of micrometastasis. Nine randomized trials were conducted on patients with confirmed locoregionally confined esophageal cancer who received preoperative CRT compared to surgery alone (30–38). Two of these studies demonstrated an improved outcome, despite the limited patient sample (30,38), whereas the remaining studies showed no survival benefits in the trimodality therapy group. Therefore, the benefits of preoperative CRT remain controversial. In addition, there is no ongoing or planned randomized study that is related to preoperative CRT for ESCC in Japan. Since 1996, preoperative CRT using 5-fluorouracil (5-FU) and cisplatin (CDDP) combined with radical surgery has been employed for the treatment of advanced esophageal cancers, with reported increased resectability, reduced incidence of local recurrence and distant metastasis and a more favorable prognosis for CRT responders (39). Additionally, we have reported that preoperative CRT in UICC stage II/III (non-T4) ESCC contributed to tumor shrinkage, leading to higher resectability and longer patient survival (40).

In the present retrospective study on patients with resectable esophageal cancer who underwent extensive radical esophagectomy, we investigated whether increased survival benefits were obtained from neoadjuvant CRT plus surgery, compared to surgery alone.

## Patients and methods

### Patients

Patients with histologically confirmed ESCC who had not undergone treatment previously were considered eligible for inclusion in the present study. Endoscopy and CT scan and/or endoscopic ultrasound examination were mandatory for determining clinical stage (II, III or IV disease) in patients with resectable disease according to the UICC TNM Classification of Malignant Tumors (41). The eligibility criteria for the present study were as follows: age <80 years, adequate organ function (white blood cell count ≥3,500, hemoglobin ≥10 g/dl, aspartate aminotransferase/alanine aminotransferase ≤2× upper limit of normal, platelet count ≥100,000/mm^3^, serum creatinine ≤1.3 mg/dl) and a performance status (Eastern Cooperative Oncology Group) of <2 at the time of admission. Eighty-eight patients were enrolled in our study. Of these, 52 patients received preoperative CRT followed by esophagectomy (Group A) and 36 patients received esophagectomy alone (Group B) between August, 1997 and June, 2011 at the Departments of Surgery of Hyogo College of Medicine (Nishinomiya, Japan) and Nara Hospital, Kinki University School of Medicine (Ikoma, Japan). Informed consent was obtained from the patients.

### Neoadjuvant CRT followed by esophagectomy

Neoadjuvant radiotherapy (1 fraction/day) was performed for 5 days per week (Monday to Friday) using a linear accelerator (Mevatron KD2; Toshiba, Kawasaki, Japan). Patients received 20 fractions of 2 Gy up to a total radiation dose of 40 Gy. The radiation field encompassed the primary tumor volume (as defined by endoscopy, esophagography and CT scans) with a 3-cm margin in each cephalad and caudal direction and 4-cm horizontal margins. If lymph node metastasis was detected using CT, the radiation field was extended to include the primary tumor and the metastatic lesions. Concurrent chemotherapy consisted of 5-FU (500 mg/m^2^/day) administration by a 120-h continuous intravenous infusion starting on day 1 and CDDP (15–20 mg/day) by a 2-hour intravenous infusion on days 1–5 and repeated after 3 weeks. Esophagectomy was planned for 4–7 weeks following completion of CRT. The majority of patients underwent thoracotomy, laparotomy and cervicotomy in order to perform esophagectomy with two- or three-field lymphadenectomy and gastroesophageal anastomosis at the left side of the neck. Radical resection (R0) was defined as the removal of the macroscopic tumors, no evidence of distant metastasis, absence of a microscopic residual tumor, free resection margins and lymphadenectomy extending beyond the involved nodes. Resection was defined as non-radical when microscopic (R1) or macroscopic (R2) residual tumor was identified according to the TNM criteria (41).

### Evaluation of response after CRT

The effects of CRT on the primary tumor and the metastatic nodes were assessed at 2–3 weeks after the completion of radiotherapy, using chest CT scanning, barium esophagography and/or upper gastrointestinal endoscopy and/or ultrasonography. The response to therapy was defined according to the criteria of the Japanese Society of Esophageal Disease (42) as follows: i) complete response (CR), defined as 100% regression of cancer; ii) partial response (PR), defined as >50% regression of the primary tumor and metastatic nodes; iii) progressive disease (PD), defined as an increase of 25% in the size of the primary tumor or metastatic nodes or the appearance of new lesions; and iv) no change (NC) defined as a decrease of <50% in the size of the primary tumor and metastatic nodes and no evidence of tumor progression. Toxicities were classified according to the National Cancer Institute Common Terminology Criteria (NCI CTC) guidelines (43).

### Esophagectomy (surgery alone)

Esophagectomy was performed through a small thoracotomy (∼10 cm) using thoracoscopy-assisted esophagectomy with two- or three-field lymphadenectomy including the upper mediastinum. The reconstruction was routinely performed using the retrosternal root and gastroesophageal anastomosis at the left side of the neck. The degree of radical resections (R) was similarly assessed according to the TNM system (41).

### Locoregional failure and distant metastasis

After the first recurrence was noted, any additional recurrence identified within 1 month was considered to have occurred simultaneously. Locoregional recurrences were defined as anastomotic recurrences or recurrences that occurred either in the mediastinum or the upper abdomen at the site of previous esophageal resection and nodal clearance. Distant recurrences were defined as hematogenous or other types (in the pleura or peritoneum). Cervical, celiac axis and para-aortic nodal metastases were classified as distant metastasis according to TNM system (41).

### Statistical analysis

The differences between the two groups (A and B) in terms of patient characteristics, postoperative complications and recurrence patterns were evaluated using the Fisher’s exact test. Overall survival (OS) was defined as the time from the date of initial treatment to patient death or to the date of the last available information on the vital status. Disease-free survival (DFS) was defined as the length of time after treatment during which no cancer was detected. Differences between the cumulative survival rates of the patient groups were calculated using the log-rank test for comparisons of the Kaplan-Meier survival curves. P<0.05 was considered to indicate a statistically significant difference. Univariate analyses were used to assess patient characteristics and other prognostic factors. The Cox proportional hazards model was used to determine differences in survival between the two treatment groups and subgroups. Statistical analyses were performed using STATISTICA software, version 06J (StatSoft, Tulsa, OK, USA) and SPSS statistics, version 16 (SPSS Japan Inc., Tokyo, Japan).

## Results

### Patient characteristics

The patient characteristics are summarized in Table I. Eighty-eight patients were evaluated in this study, including 52 patients in Group A (neoadjuvant CRT+surgery) and 36 patients in Group B (surgery alone). The tumors were histologically confirmed as ESCCs. No statistical differences were observed in age, male/female ratio, location of primary tumor, lymph node metastasis or clinical stage. As regards the depth of tumor invasion, there was a tendency for deeper invasion in Group A compared to that in Group B patients (P<0.001). R0 resection was performed in 42 patients in Group A and the patients in Group B. Ten patients in Group A underwent R1 resection. Patients with positive cervical and/or celiac nodes (M1a, M1b) were classified as clinical stage IV. One patient in Group A had a double cancer of the esophagus and lower pharynx. She received radiotherapy to the neck preoperatively, at a total dose of 60 Gy, in order to preserve the pharynx and larynx.

### Response to neoadjuvant CRT and toxicity

The clinical response of the primary tumor and the metastatic nodes is provided in Table II. Lymph node metastasis was observed in 18 patients by CT scans or upper gastrointestinal ultrasonography. The patients with NC and PD for metastatic nodes had celiac or neck lymph node metastasis, respectively. At the radiologists’ suggestion, these fields were excluded from the primary radiation field to avoid postoperative complications. The clinical response (CR+PR) to neoadjuvant CRT for the primary tumor and the metastatic nodes was 86.5 and 44.4%, respectively. The collective clinical response of the primary tumor and metastatic nodes was 80.8%. Major toxicology profiles were summarized in medical records.

Leukocytopenia exceeding grade 3 was observed in 21.1% of patients, grade 1/2 general fatigue was observed in 30.8%, grade 1/2 nausea in 28.9%, grade 2/3 loss of appetite in 23.1% and grade 2 liver dysfunction in 3.8% of the patients, respectively. There was no reported CRT-related mortality.

### Surgery and postoperative complications

Eighty-five patients (51 patients in Group A and 34 in Group B) underwent esophagectomy via right thoracotomy with two- or three-field lymphadenectomy in both groups. One patient in Group A underwent lower esophagectomy with proximal subtotal gastrectomy via left thoracotomy and jejunal reconstruction was performed. Furthermore, one patient in Group A received a total esophagectomy with laryngectomy. Additionally, one patient in Group A received ileocecal replacement since he had previously undergone gastrectomy for early gastric cancer. Two patients in Group B underwent lower esophagectomy with proximal subtotal gastrectomy via left thoracotomy and jejunal reconstruction was performed. Postoperative complications from medical records are summarized in Table III. Leakage following esophagogastrostomy was observed in 4 patients (7.5%) in Group A, a lower incidence compared to Group B (P=0.027). Two patients in Group A underwent additional surgery to restore the continuity of the alimentary tract. One patient received jejunal interposition between the gastric tube and the neck of the esophagus and another patient received skin flap transplantation of the latissimus dorsi muscle.

### Pathological response of the primary tumor

Sixteen out of the 52 patients that received neoadjuvant CRT (30.8%) showed no residual tumor in the resected esophagus, which was compatible with pathological CR.

### Recurrence pattern

Comparisons of incidence and type of disease recurrence between the two treatment groups are provided in Table IV. The incidence of simultaneous locoregional and distant recurrence was significantly higher in Group B compared to that in Group A (P=0.0474).

### DFS and OS

**Figure 1.**
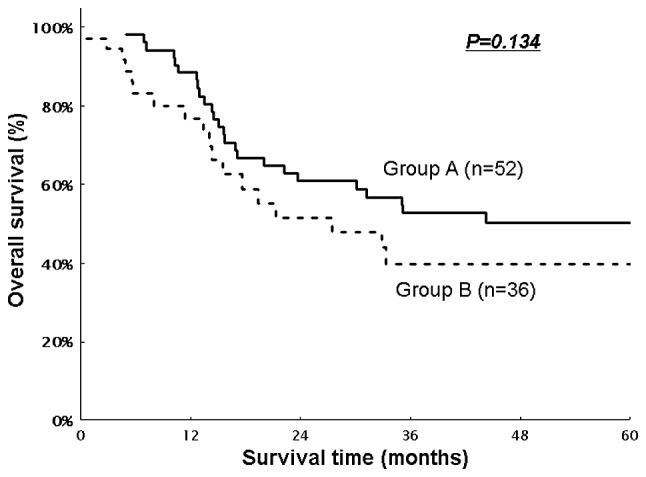
Comparison of overall survival between the neoadjuvant chemoradiotherapy (CRT) (Group A) and surgery alone (Group B) groups in stage II–IVa esophageal cancer patients.

**Figure 2.**
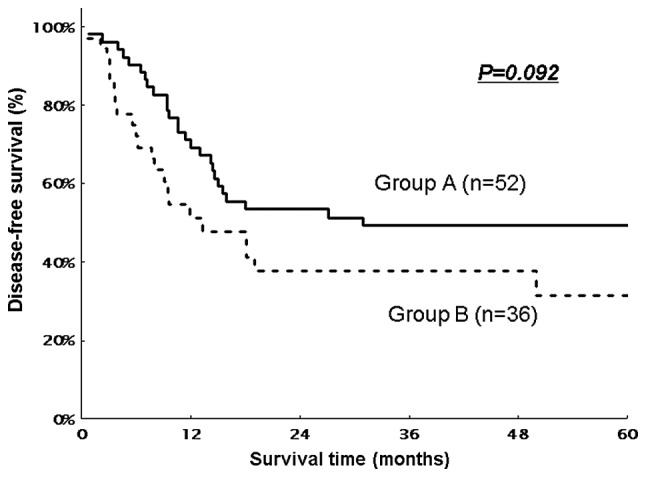
Comparison of disease-free survival between the neoadjuvant chemoradiotherapy (CRT) (Group A) and surgery alone (Group B) groups in stage II–IVa esophageal cancer patients.

The median follow-up period was 44.8 months in Group A and 24.6 months in Group B. The OS for Groups A and B, including hospital deaths, is shown in Fig. 1. The median survival time (MST) was not achieved in Group A and was 27.4 months in Group B. The estimated 3- and 5-year OS rates were 52.7 and 50.3%, respectively, in Group A and 39.9 and 39.9%, respectively, in Group B. There was no significant difference between the two groups (P=0.134). The DFS in the two groups is shown in Fig. 2. The MST was 30.97 months in Group A and 13.37 months in Group B. The estimated 3- and 5-year DFS rates were 49.4 and 49.4%, respectively, in Group A and 37.7 and 37.4%, respectively, in Group B. Group A exhibited a tendency for a higher DFS rate compared to Group B, although there was no significant difference between the two groups (P=0.092). In the subgroup analysis of the patients with stage II/III disease, Group A exhibited a significantly improved OS rate compared to Group B (5-year OS, 59% for Group A vs. 39.9% for Group B, P=0.043). Similarly, a higher DFS rate was observed in Group A compared to Group B (5-year DFS, 57.2% for Group A vs. 31.4% for Group B, P=0.025).

### Subgroup analysis

**Figure 3.**
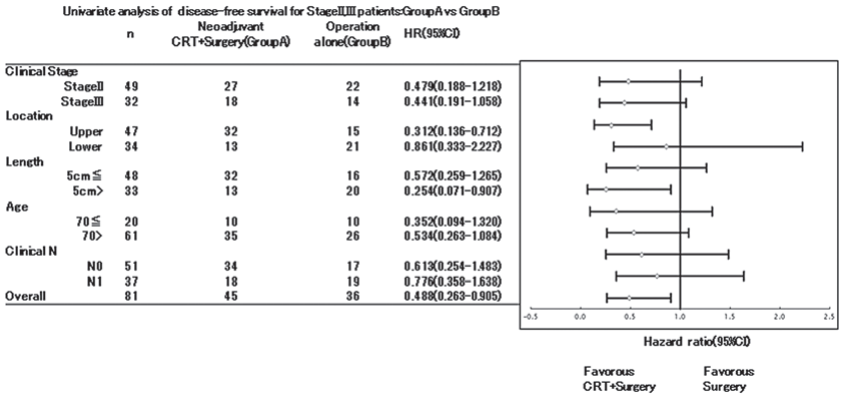
Univariate analysis of disease-free survival between Groups A and B in stage II/III esophageal cancer patients.

The results of the subgroup analysis for OS and DFS according to clinical stage, lymph node status, tumor depth of invasion, tumor location and resectability of stage II/III patients are shown in Table V. Patients with N0 tumors that received neoadjuvant CRT (Group A) had a significantly prolonged DFS compared to patients with N1 tumors and Group B patients. As regards OS, Group A exhibited a significantly prolonged survival rate and patients with N0 tumors showed a tendency for improved survival (P= 0.057). Subgroup analysis regarding DFS is shown in Fig. 3. Patients with tumors located in the upper esophagus and tumors ≥5 cm in length exhibited a higher survival rate in Group A compared to Group B [hazard ratio (HR), 0.312 and 0.136–0.712, respectively, for tumor location, HR, 0.254 and 0.071–0.907, respectively, for tumor length].

## Discussion

In this retrospective study, we demonstrated that neoadjuvant CRT with 5-FU and CDDP conferred an increased survival benefit on patients with resectable stage II/III esophageal cancer, compared to surgery alone. As regards preoperative CRT, we have already shown that CRT in stage II/II (non-T4) patients contributed to tumor shrinkage, leading to higher resectability and longer survival (40). In this retrospective study, we only evaluated patients with resectable tumors, including T4 patients downstaged by neoadjuvant CRT. As shown in Table II, the clinical response rate for the primary tumor and the metastatic nodes following CRT exceeded 80%. Therefore, neoadjuvant CRT proved to be effective for stage II/III esophageal cancer patients. Over the last few decades, surgical techniques and perioperative management, as well as the overall prognosis of esophageal cancer patients undergoing surgical resection, have significantly improved (13,26,44). In Japan, three-field lymph node dissection is currently considered acceptable as a standard therapy for advanced esophageal SCC. However, the survival benefit of three-field lymph node dissection remains controversial (45–47). There have been no randomized phase III trials on two- or three-field lymph node dissection for ESCC in Japan. At present, we omit the procedure of neck lymph node dissection when there is no lymph node enlargement detected on preoperative CT scans. Our data demonstrated that the 5-year OS rate for surgery alone in stage II/III patients was 39.9%, whereas the current survival rates of pathological stage IIA, IIB and III patients, classified according to UICC criteria, are reported to be 51.5, 34.0 and 19.8%, respectively, according to the comprehensive registry of esophageal cancer in Japan (48). CRT for esophageal cancer has been developed mainly in the United States, since an RTOG study revealed that clinical benefits from CRT were superior to those from radiotherapy alone in the patients with localized carcinoma of the esophagus (49,50). There have been two contradictory reports on stage II/III patients regarding the comparison of trimodality therapy compared to surgery alone (33,38). Tepper *et al* (38) evaluated patients in a neoadjuvant CRT group and demonstrated a survival advantage compared to surgery alone, supporting trimodality therapy as a standard of care for the patients with stage IIa-III esophageal cancer; however, this trial included a limited number of patients and the 5-year survival rate was significantly lower (39% in the trimodality group vs. 10% in the surgery only group) (38). However, Apinop *et al* failed to demonstrate a clinical advantage due to trimodality therapy compared to surgery alone in 69 patients with stage IIb-III ESCC. This study also included a limited patient sample and the 5-year survival rate was low (24% in the trimodality group vs. 10% in the surgery alone group) (33). Furthermore, neither study addressed the details of operative procedures, which may be different in Japan regarding lymph node dissection. Therefore, it is difficult to assess the efficacy of trimodality therapy in the same dimension. Burmeister *et al* (37) reported that trimodality therapy improved DFS, unlike OS, in patients with stage I–III ESCC, excluding adenocarcinoma (HR: 0.47 and 0.25–0.86, respectively) (37). This subset analysis has encouraged us to continue trimodality therapy for ESCC in Japan. We also reported an improved prognosis, especially for patients with tumors located higher and <5 cm in diameter. However, the efficacy of CRT using our current regime may not suffice for tumors >5 cm in diameter.

As regards the pattern of recurrence following surgical resection, we demonstrated a more frequent simultaneous locoregional and distant recurrence in patients after surgery alone. Neoadjuvant CRT has been reported to control tumor micrometastasis and to inhibit locoregional and distant metastasis (51). Locoregional recurrence and distant metastasis after radical esophagectomy with two- or three-field lymph node dissection have been found to vary from 11.3 to 32.6% and the incidence of simultaneous locoregional recurrence and distant metastasis has been reported to range from 1.1 to 13.9% (19–25). The major complications following esophagectomy with radical lymph node dissection were anastomotic leakage, recurrent nerve palsy and respiratory complications. Previous Japanese studies have indicated hospital mortality in 2.2–12.3% of the patients who underwent two- or three-field lymph node dissection, anastomotic leakage in 11–39%, recurrent nerve palsy in 9–76% and respiratory complications in 8–32% (26,27,52,53). The incidence of postoperative complications after surgery alone in our study were in accordance with these data. Even when CRT was administered in combination with esophagectomy with extended lymph node dissection, the incidence of postoperative complications did not increase in our study. In Japan, neoadjuvant chemotherapy (CDDP plus 5-FU) followed by esophagectomy is currently considered the standard treatment for stage II/III esophageal cancer, although this was considered disputable by previous randomized studies (54–56). We strongly recommend that the the efficacy of CRT is objectively evaluated in Japan by additional JCOG studies.

In conclusion, treatment with neoadjuvant CRT consisting of 5-FU and CDDP did not contribute to a better prognosis in patients with resectable ESCC. However, it may be beneficial for patients with stage II/III disease. Additional large prospective randomized controlled trials involving preoperative CRT are required to elucidate whether this treatment may improve the prognosis of ESCC patients.

## Figures and Tables

**Table I.** Clinicopathological characteristics.

Variables	Group A	Group B	P-value
Age (years)			
Mean	60.11	64.6	NS
Gender			
Male	42	32	NS
Female	10	4	
Location of primary tumor			
Upper esophagus	7	2	NS
Middle esophagus	29	13	
Lower esophagus	16	19	
Abdominal	0	2	
Depth of tumor invasion			
T1b	0	1	<0.001
T2	2	14	
T3	34	21	
T4	16	0	
N-classification			
N0	34	17	NS
N1	11	19	
M1a	2	0	
M1b	5	0	
Clinical stage			
II	27	22	NS
III	18	14	
IV	7	0	

NS, non-significant.

**Table II.** Effects of preoperative CRT on the primary tumor and the metastatic nodes.

Response	Primary tumor	Metastatic nodes	Clinical response rate (Primary tumor and metastatic nodes)
CR	16	4	14
PR	29	4	28
NC	7	8	8
PD	0	2	2
Response rate	86.5%	44.4%	80.8%

CRT, chemoradiotherapy; CR, complete response; PR, partial response; NC, no change; PD, progressive disease.

**Table III.** Postoperative complications in the two groups.

	Group A	Group B	P-value
Blood loss (ml), mean	528	684	NS
Respiratory failure (%)	5.7	13.8	NS
Anastomotic leakage (%)	7.5	25	0.027
Recurrent nerve palsy (%)	3.8	5.25	NS
30-day mortality (%)	0	2.78	NS
Hospital death (%)	1.9	8.3	NS

**Table IV.** Site of recurrence in 88 esophageal squamous cell carcinoma patients.

Site	Group A No. (%)	Group B No. (%)	P-value
Locoregional failure	4/52 (7.69)	5/36 (13.9)	NS
Distant metastasis	15/52 (28.8)	7/36 (19.4)	NS
Local and distant simultaneously	2/52 (3.85)	6/36 (16.7)	0.0474

NS, not significant.

**Table V.** Univariate analysis of survival for stage II/III esophageal cancer patients.

Variables	No.	Disease-free survival	P-value	Overall survival	P-value
		Hazard ratio (95% CI)		Hazard ratio (95% CI)	
CRT+surgery vs. surgery alone	45/36	0.488 (0.263–0.905)	0.023^a^	0.516 (0.269–0.989)	0.046^a^
Men vs. women	68/13	0.600 (0.235–1.530)	0.285	0.723 (0.282–1.858)	0.501
Age (70> vs. ≥70) (years)	20/61	0.792 (0.429–1.463)	0.456	1.120 (0.580–2.163)	0.735
Clinical N0 vs. N1	51/30	0.452 (0.245–0.836)	0.011^a^	0.532 (0.278–1.018)	0.057
^b^Tumor location (upper vs. lower)	47/34	1.208 (0.652–2.239)	0.548	1.316 (0.688–2.514)	0.406
Tumor length ≥5 vs. <5 (cm)	48/33	1.197 (0.638–2.262)	0.579	1.546 (0.766–3.040)	0.229

a Statistically significant;

b upper, tumor located above the bifurcation and mid-esophagus; lower, tumor located in the lower and abdominal esophagus. CRT, chemoradiotherapy; CI, confidence interval.
